# Understanding the errors made by artificial intelligence algorithms in histopathology in terms of patient impact

**DOI:** 10.1038/s41746-024-01093-w

**Published:** 2024-04-10

**Authors:** Harriet Evans, David Snead

**Affiliations:** 1 Histopathology Department, University Hospitals Coventry and Warwickshire NHS Trust, Coventry, UK; 2 Warwick Medical School, University of Warwick, Coventry, UK; 3 Department of Computer Science, University of Warwick, Coventry, UK

**Keywords:** Pathology, Outcomes research

## Abstract

An increasing number of artificial intelligence (AI) tools are moving towards the clinical realm in histopathology and across medicine. The introduction of such tools will bring several benefits to diagnostic specialities, namely increased diagnostic accuracy and efficiency, however, as no AI tool is infallible, their use will inevitably introduce novel errors. These errors made by AI tools are, most fundamentally, misclassifications made by a computational algorithm. Understanding of how these translate into clinical impact on patients is often lacking, meaning true reporting of AI tool safety is incomplete. In this Perspective we consider AI diagnostic tools in histopathology, which are predominantly assessed in terms of technical performance metrics such as sensitivity, specificity and area under the receiver operating characteristic curve. Although these metrics are essential and allow tool comparison, they alone give an incomplete picture of how an AI tool’s errors could impact a patient’s diagnosis, management and prognosis. We instead suggest assessing and reporting AI tool errors from a pathological and clinical stance, demonstrating how this is done in studies on human pathologist errors, and giving examples where available from pathology and radiology. Although this seems a significant task, we discuss ways to move towards this approach in terms of study design, guidelines and regulation. This Perspective seeks to initiate broader consideration of the assessment of AI tool errors in histopathology and across diagnostic specialities, in an attempt to keep patient safety at the forefront of AI tool development and facilitate safe clinical deployment.

## Introduction

Histopathology, along with other image-based diagnostic specialities such as radiology, are seeing significant development of artificial intelligence (AI) tools. In histopathology the potential uses of AI include diagnosis, prognostication, workflow applications, and education^1^. In diagnostic terms, AI could increase diagnostic accuracy and efficiency^1,2^ which is invaluable in the face of a rising workload and a widespread lack of pathologists^3,4^.

However, AI tools and the machine learning (ML) algorithms that form them, are not infallible and perfect accuracy is unlikely to be achievable^5^. Therefore, the introduction of AI will bring not only the aforementioned benefits, but also the pervasive issue of AI tool errors. A report by the European Parliamentary Research service identified patient harm from AI errors as one of the major risks arising from the introduction of AI into healthcare^6^.

Currently AI tool errors are predominantly reported in terms of technical performance metrics, which although are undoubtably important to the safe assessment of a tool, do not adequately explain how these misclassifications translate into impact on patients^7,8^. The consequences of AI tool errors are vital to understand and report because they have the potential to cause profound and harmful effects on people^7,9^. The literature highlights that transparency and validation of tools in terms of their impact on clinical outcomes is essential to build trust in AI^2^ but such reporting of the clinical impact of AI tool errors is currently lacking in histopathology and other specialities. This is likely contributing to the described “implementation chasm” between AI tool development and clinical use^5^. This Perspective aims to broaden the horizons of how we consider and report errors made by AI tools, suggesting a more clinical focus is greatly warranted.

## Definitions of error

### Clinical diagnostic errors

The World Health organisation states that “A diagnostic error emerges when a diagnosis is missed, inappropriately delayed or is wrong”^10^. Another definition describes diagnostic errors as “the failure to (a) establish an accurate and timely explanation of the patient’s health problem(s) or (b) communicate that explanation to the patient”^11^.

It is key to state that not all medical errors result in patient harm^12^. Some errors have potentially serious consequences such as death or injury, however, others may not even cause a noticeable impact. This has led to the use of the term ‘adverse event’ to describe medical errors that result in patient harm^12^.

### AI tool errors

ML models are trained on large amounts of data to learn patterns that enable the model to make predictions. In histopathology diagnostics these predictions can include identification and classification of entities, grading of tumours and quantification tasks that encompass roles currently assigned to human pathologists^13^, however, at the ML model level these remain simply classifications, and any errors are misclassifications between one group and another. When assessing a ML model’s performance, the model classifications are compared to a chosen ground truth, usually a pathologist’s diagnosis, again framing errors as simply misclassifications.

In general, the deep neural networks that form the basis of many AI tools are trained to minimise errors but assume that all types of error are equal. This is termed being ‘insensitive to impact’^14,15^. This assumption is vastly incorrect in healthcare, where the consequences of different misclassifications could have hugely different outcomes for patients in terms of treatment decisions and prognosis.

We therefore need to translate these AI tool errors or misclassifications, into meaningful error information that is understandable in the clinical context.

## Current assessment of histopathologist errors

A literature review from the College of American Pathologists summarising 116 studies on diagnostic errors in pathologists found the median discrepancy rate between the primary report and secondary pathologist review was 18.3%, with a rate of major discrepancies of 5.9%^16^. They acknowledge that “not all errors are alike”, and across the review found 81 different definitions of major or significant diagnostic discrepancy, highlighting that although studies aimed to quantify error severity, standardised definitions are lacking.

Several studies give detailed pathological information about pathologist errors, such as Oxley et al. who assessed for errors in 4192 prostate core biopsies and found that the diagnostic category changed in 146 cases. They quantified the misdiagnoses, for example 32 cases were upgraded to adenocarcinoma (from previous diagnosis of suspicious for malignancy (5 cases), high-grade prostatic intra-epithelial neoplasia (5 cases) and benign (22 cases)). Additionally, they discussed reasons for these errors, such as small tumour area and rare morphology^17^. An example of a study quantifying not only the number and type of errors, but also their clinical impact is Kronz et al. who, when considering the clinical impact of lesions missed on prostate biopsies, found that although they identified 87 errors across 3251 patients, only 15 of these misses, if found, would have resulted in a definitive change in care, and 17 a possible change in care (e.g., bilateral vs unilateral cancer)^18^.

Importantly, because the pathologist’s diagnosis forms the basis of a report to guide the clinician, but does not automatically result in a defined outcome, the clinical impact of a diagnostic error is also determined by the clinician’s response to the report. Highlighting this is work by Raab et al. who reported diagnostic discrepancies by review of histology and cytology specimens and found that in non-gynaecological cases, 5% had errors that were near misses, meaning the clinician intervened before harm occurred or did not act on the incorrect diagnosis^19^.

## Current assessment of AI tool errors in histopathology

In order to understand the current approach taken in studies on AI tools in histopathology, a literature search was conducted through PubMed, Medline, Embase, Web of Science and Google scholar using terms: ‘Pathology’ or ‘Histopathology’ AND ‘Artificial intelligence’ OR ‘Machine learning’ OR ‘Neural network’ OR ‘Computer-assisted diagnosis’ AND ‘Risk’ (including risk assessment/evaluation/reduction/management/ factor/patient risk) OR ‘Error’ (including diagnostic error or medical error).

This search found no papers that specifically discussed the measurement or approach to errors in AI tools in histopathology. Instead, it highlighted that the literature consists of a vast number of small studies evaluating a single AI tool performing a narrowly defined task. In such studies, the technical accuracy of tools is well documented, using a wide range of robust performance metrics including sensitivity and specificity, positive and negative predictive values and area under the receiver operating characteristic curve, or area under the precision-recall curve^20^. Calculating the area under these curves each gives a single powerful metric that measures a ML tool’s ability to differentiate between two binary groups^21^. It is, of course, fundamental that a tool can classify entities well, but without understanding, in clinical terms, of the implications of different classifications, or misclassifications, this only gives a report of the volume of errors, not their severity.

These metrics do allow a rudimentary understanding of an AI tool’s error profile in terms of its false negative and false positive results, but the implications of different false negatives and positives are likely to vary. With a screening tool designed to detect a region of interest, for example cancer detection, false positive results where the slide is normal but is flagged as abnormal will need to be reviewed by a pathologist, potentially creating additional workload for the pathologist and increasing reporting time. This is likely to be considered less costly than false negative results, where the slide contains pathology but is labelled as negative by the tool. The risk here is that failure to recognise the region could mean that the patient does not receive the correct diagnosis, potentially missing out on treatment and succumbing to disease progression.

These metrics are essential, allowing standardised reporting and comparison between tools and should always remain the foundation of any AI tool assessment, however, we argue that they alone are not sufficient.

## Comprehensive approaches to reporting AI tool errors

In order to develop an appropriate strategy for error management of AI tools in pathology we need to consider the risks of errors made at a level beyond simply the algorithm output, but rather at the level of the pathology diagnosis or, more holistically, in terms of clinical outcome.

### Reporting of errors in terms of the histopathological diagnosis

Having pathological information about each error, for example the entity concerned and other relevant information such as the size and location, would allow some understanding of error severity based on the pathological understanding of different diseases. Details of the pathological nature of errors is seen in some studies but is not widespread practice and it lacks consistency, in contrast to studies on human pathologists where this information is widely provided.

Graham et al. published their colon biopsy screening algorithm, which classifies entities into normal and abnormal, and studied both false positive and false negative errors^22^. Analysis of false positive cases with the highest confidence scores identified that the algorithm had actually correctly classified several of these cases but there has been mislabelling errors in the dataset, thus meaning a slight improvement in the algorithm’s performance. Analysis of the false negatives highlighted the pathological entities where the algorithm performance was weak, such as lymphocytic and collagenous colitis^22^. This demonstrates how pathological interpretation of the errors made can have several benefits: aiding understanding of where the tool would struggle in practice, setting out further training requirements and even refining the original results.

An important point is highlighted in a study on Paige Prostate-AI for the detection of prostate carcinoma and atypia. In the study, several of the false negative cores were instances of glandular atypia. The authors note that although these lesions are not actionable from a urologist’s perspective, they are important to detect from the pathologist’s stance because they can prompt further work such as further levels or immunohistochemistry, which then may lead to the detection of further disease^23^. This demonstrates how knowing the pathological nature of errors made has unique merit because this can shape pathologist’s actions and potentially the final diagnosis.

The use of standardised pathological terms and entities would allow pathologists and clinicians to understand the nature of each misclassification made. Furthermore, there is merit in categorising errors, such as into major and minor errors, as seen in error rates in human pathologists, although this currently lacks standardisation and so a collaborative approach would be required to define such groups.

### Reporting of errors in terms of the clinical impact

As discussed, not all errors in medicine cause harm; some errors go completely unnoticed but others are serious adverse events. Therefore, to understand the true consequences that AI tool errors have, we need to consider the potential clinical consequences of each misclassification.

Separating errors into false positives and false negatives is the most simplistic distinction of errors based on potential impact, however the severity of different types of false positives or negatives are not equal. For example, in a colon biopsy screening tool, a false negative result in a patient with adenocarcinoma is unlikely to be considered the same clinically as a missed mild chronic inflammation, with the former potentially being a serious adverse event, and the latter potentially having no patient impact. Discussions on clinical impact require clinical team input and can be complex, requiring knowledge of the care pathways for different results. One example is seen in the Paige Prostate-AI study cited previously, where clinical impact is considered by reporting that in 4/5 missed adenocarcinoma cores the patient had cancer detected in another core and that in the fifth case the patient was known to have low volume disease and was under routine surveillance already, thus these misses were unlikely to significantly impact management^23^.

With more sophisticated algorithms, more complex clinical questions arise. For example, with algorithms that grade cancers, is the risk of harm the same for up-grading, compared to down-grading a tumour? If, or when, algorithms are introduced that diagnose specific entities (rather than just normal versus abnormal), we will be considering the impact of one diagnosis compared to another.

There is a need for standardised terminology to report on the clinical impact of AI tool errors. We suggest this could be done either by considering the severity of patient impact (e.g., none, mild, moderate, severe) or the type of impact on patient care (e.g., no change in care, delayed diagnosis, missed/delayed investigations, missed/ delayed treatment).

**Fig. 1:**
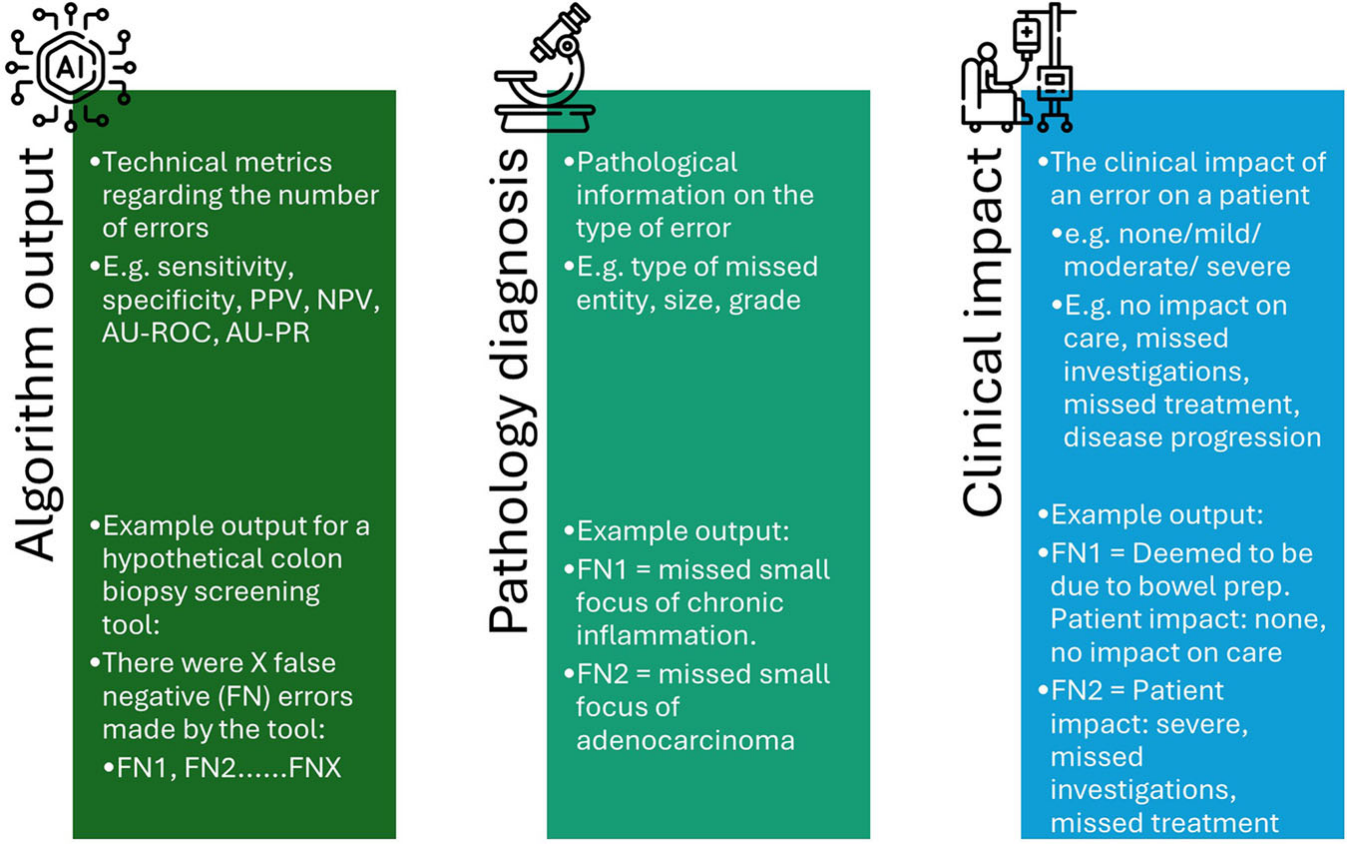
The different stages at which to consider the errors made by an AI diagnostic algorithm in pathology. This figure demonstrates how errors can be considered in terms of technical metrics, the pathological information or the clinical impact, with a hypothetical example of two different false negatives from a colon biopsy screening algorithm. (FN- False negative, PPV- positive predictive value, NPV- negative predictive value, AU-ROC – area under the receiver operating characteristic curve, AU-PR- area under the precision-recall curve). Icons used in image from Freepik from Flaticon.com.

**Fig. 2:**
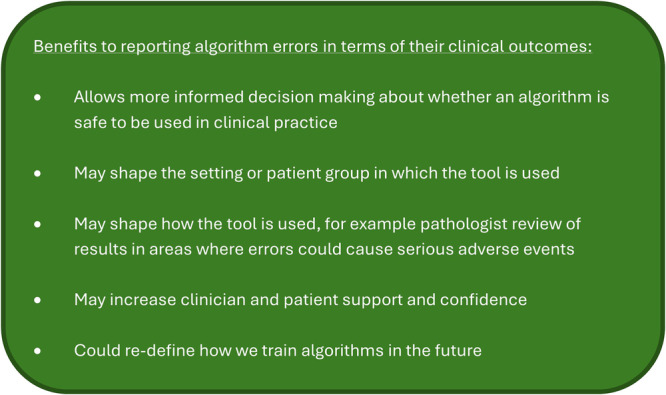
The potential benefits of reporting AI pathology tool errors in terms of clinical impact. This is not an exhaustive list but details some of the most important reasons why a more holistic consideration of errors is beneficial.

Although there is likely to be debate about the relative harms of different errors^24^, if we agree that there are differences, then simply considering the technical properties of an algorithm and the volume of errors is an inadequate strategy. Figure 1 summarises the different levels at which algorithms can be assessed, and Fig. 2 proposes some of the benefits of a holistic, clinical consideration of errors.

## Parallels to radiology

In radiology, a similar diagnostic speciality that has seen a much greater influx of AI tools, interesting work demonstrates that assessing AI tools can be done at multiple hierarchical levels.

In 1991 Fryback and Thornbury stated that when evaluating any diagnostic imaging, it is important to go beyond simply the quality and accuracy of the radiological examination^25^. They note that because any diagnostic imaging process sits within wider clinical and societal realms, there are numerous levels at which to assess its efficacy. They proposed a hierarchical six tier system to assess diagnostic imaging tools, which was then adapted by van Leeuwen et al. in order to specifically appraise AI tools^26^. An adapted version is seen in Table 1.

**Table 1:** Hierarchical model to assess the efficacy of AI software in the diagnostic imaging process adapted from van Leeuwen et al.^26^ and Fryback and Thornbury^25^

Level	Consideration
Level 1t: Technical efficacy	What is the technical usability of the software? For example reproducibility, error rate
Level 1c: Potential clinical efficacy	What is the correlation between this software and other methods used such as biomarker studies?
Level 2: Diagnostic accuracy efficacy	What is the standalone performance of the software? Sensitivity, specificity, Area under the receiver operating characteristic curve
Level 3: Diagnostic thinking efficacy	What is the impact of the software on the diagnosis? Diagnosticians performance with and without AI Any changes in diagnostic judgement?
Level 4: Therapeutic efficacy	What is the impact of the software on patient management decisions? Effect on treatment and follow up
Level 5: Patient outcome efficacy	What is the impact of the software on patient outcomes? Effect on quality of life, morbidity, survival
Level 6: Societal efficacy	What is the impact of the software on society? Economic effect

van Leeuwen et al. evaluated the literature on 100 CE-marked AI products in radiology, appraising them against this model. They found that 64/100 products had no peer reviewed evidence of safety, with the other 36 products had a combined evidence base of 237 studies. Of these, 65% only focused on diagnostic accuracy (level two). Only 18 products had studies that discussed evidence at level three or higher, meaning that the vast majority of these products had no available data on their potential clinical impact in terms of either the clinical benefits or harms^26^. As noted in the paper discussion, the level of efficacy assessment required for a tool varies according to its intended use, for example it is likely to be lower if the tool is only intended to aid the clinician (e.g., by performing a measurement task for a radiologist) than if it is an independent diagnostic tool, however the lack of clinical consideration across approved tools is surprising. If we apply this model to pathology, the algorithmic output measurements we currently see reported for AI tools equate to level two, but as discussed there is benefit in including wider assessment of accuracy, namely level three: how does the AI tool affect the pathological diagnosis?, and level four: how does the AI tool impact patient management decisions? In the future, levels five and six could also be considered, although there could be significant time and cost involved.

## Future directions

### Reporting of errors in the literature

We argue that for any AI diagnostic tool that is nearing the clinical realm there should be an assessment of the expected clinical impact of errors to supplement the technical metrics reported, in order to aid pathologists, clinicians, healthcare organisations and patients to gain a wider understanding of the errors made.

Furthermore, as workloads increase and the pathologist shortages continues, it is increasingly likely that AI tools will assist or replace histopathologists in certain diagnostic tasks. We therefore will need to compare the errors made by AI tools with the current standard of care which is human pathologists. This relies on a standardised reporting of the clinical implications of errors to facilitate a cross modality comparison of safety.

In order to achieve this there must be high-quality pathology and clinical data collection and interpretation built into study design, and tool development and analysis conducted by multidisciplinary teams.

This is a call to action for researchers working on AI diagnostic tools, in histopathology and other specialities, to allow time, finances, and personnel in study plans to consider the broader consequences of AI tool errors. This should be fruitful for teams, in terms of refining study results, and increasing transparency for all concerned.

### Guidelines

Guidelines are essential to support and standardise this process but, as stated by Wismüller and Stockmaster, there is a current lack of guidance on “objectively evaluating AI systems with regards to clinically meaningful performance metrics”^8^.

Several AI specific guidelines do exist for the evaluation of various trial types; including CONSORT-AI (Consolidated Standards of Reporting Trials–Artificial Intelligence) for randomised controlled trials, SPIRIT-AI (Standard Protocol Items: Recommendations for Interventional Trials–Artificial Intelligence) for clinical trials and STARD-AI (Standards for Reporting of Diagnostic Accuracy Study- AI) for diagnostic test studies^27–29^. These helpfully provide checklists of the minimum requirements that should be reported for AI interventions and state that investigators should provide an analysis of error cases^27–29^. However, there is currently no specification that this includes a clinical assessment of errors, although this would be the ideal place to introduce such requirements and could be incorporated into future iterations.

### Prospective and randomised control trials

Most trials on AI diagnostic tools to date are retrospective in nature, often in an artificial setting or using datasets that have been filtered, and so are not representative of real data in clinical practice^2^. Furthermore, in practice, all AI tools will interact with humans to some degree (even those deemed autonomous require humans for data input and interpretation of some results), and so studying AI tools in isolation means it is impossible to truly measure the tools performance in practice. Prospective and randomised control trials allow for inclusion of human factors and allow factors such as automation bias to be studied, as well as collection of more clinically relevant outcome data.

### Regulation

Regulatory incentives are also essential to drive a shift in reporting practices. Interestingly, Gerke et al. suggest that regulators such as the Food and Drug Administration and the Medicines and Healthcare products Regulatory Agency currently regulate medical products and devices, but that to truly ensure safe practice, they must evaluate the entire system, in what they call the “systems approach”^30^. They argue that performance in an artificial testing environment is not suitable, giving an incomplete study of the risks (and benefits) of AI tools when used in practice and not allowing for the impact of human-tool interactions^30^. Although a full “systems approach” is unfeasible^30^, a wider view incorporating human interaction with AI tools, and an assessment of errors in terms of their clinical consequences could be possible.

### Training machine learning models based on the clinical outcomes of errors

As our understanding of the clinical impact of different errors increases, there may be the option of building this knowledge into algorithm development, making algorithms themselves safer and tackling the problem of ML algorithms being insensitive to impact. Santana et al. have proposed a novel method for DNN classifiers that considers risk during the training and verification stages, allowing factors such as the risk of each specific misclassification, and the likelihood of them occurring to be built into the model itself during development^14^.

## Conclusion

This perspective highlights the importance of translating the misclassification errors made by AI diagnostic tools in histopathology into the pathological and clinical realms, to understand how AI tool errors impact patients. Although this is currently only done sporadically and in an unstandardised manner, a concerted effort to make this routine practice would improve AI tool safety, transparency and facilitate their introduction to clinical practice.
